# The relationship between basic psychological needs satisfaction and university students’ academic engagement: The mediating effect of emotional intelligence

**DOI:** 10.3389/fpsyg.2022.917578

**Published:** 2022-12-01

**Authors:** Hongxia Chen, Morning Hon Zhang

**Affiliations:** ^1^School of Marxism, Zhoukou Normal University, Zhoukou, Henan Province, China; ^2^Institute for Education Science, Zhoukou Normal University, Zhoukou, Henan Province, China

**Keywords:** academic engagement, positive psychology, basic psychological needs, emotional intelligence, use of emotion

## Abstract

**Introduction:**

Basic psychological needs satisfaction (BPNS) and Emotional intelligence (EI) have been underscored as helpful psychological constructs in explaining academic engagement. However, the joint interaction of BPNS with EI abilities to explain academic engagement has not been tested. Therefore, the present study aimed to investigate the interactive role of BPNS with EI abilities in the prediction of academic engagement in a sample of Chinese university students.

**Methods:**

A questionnaire survey was administered to a sample of 466 university students. The data were analyzed using the SPSS (version 21.0) software. The first analysis consisted of descriptive statistics (including mean and standard deviation) and Pearson’s correlations among BPNS, EI, and academic engagement. Through structural equation modeling (SEM), direct and indirect effects were calculated.

**Results:**

The results showed that BPNS was positively associated with academic engagement and that only the Use of emotion dimension of EI mediated these associations.

**Discussion:**

These results suggest that important interventions incorporated with BPNS and EI abilities, especially the use of emotion ability, may be performed to promote university students’ academic engagement.

## Introduction

### Academic engagement in the current situation

As a psychologically satisfying mental state, academic engagement refers to a persistent affective and cognitive state of contentment toward studying and learning, rather than a momentary and specific condition (Salanova et al., 2010; Casuso-Holgado et al., 2013; Chen et al., 2021). Extensive research literature has indicated that academic engagement is a key facilitator of higher academic achievements, and also can effectively reduce the levels of dropout rates (e.g., Newmann et al., 1992; Chipchase et al., 2017; Kwon et al., 2018). Recently, facilitating engagement has also been revealed to reduce the adversarial impact of sociodemographic predictors on student performance (Lei et al., 2018). So far, research have showed that both personal variables and social-contextual variables could influence the student’ academic engagement (Patrick et al., 2007; Wu et al., 2010; Stoeber et al., 2011; King and Ganotice, 2014; Kilday and Ryan, 2019; MacCann et al., 2020; Virtanen et al., 2020; García-Martínez et al., 2022; Luo and Luo, 2022; Saleem et al., 2022).

Positive psychology, which is a rising field in psychology, has given us new insights into the personal and contextual variables which influenced academic engagement (Carmona-Halty et al., 2021; Dewaele and Li, 2021; Kang et al., 2021; Luthans et al., 2021; Saleem, 2022). Among the scientific existing literature, two theories need to be noticed particularly. One is the self-determination theory (SDT), which emphasizes the importance of organic interaction between internal individual factors and external social-contextual factors (Ryan and Deci, 2017). As such, only if the influence of external social-contextual factors is transformed into individual intrinsic factors through autonomous motivation, academic engagement could be sustainable and effective. The other is the demands-resources theory (JD-R), which concerns the extent to which people apply their personal resources in their everyday life (Schaufeli and Taris, 2014). According to JD-R, some personal psychological resources could influence performance by affecting engagement. For example, adaptability, compassion, mindfulness, psychological capital, self-concept, etc., being personal resources, could create a motivational process leading to academic engagement, and, in turn, improve academic achievement and students’ life satisfaction (e.g., Armenteros et al., 2021; Carmona-Halty et al., 2021; Putwain et al., 2021).

Based on these two theories, recently, a growing body of literature has been focusing on the simultaneous effects between the personal and social-contextual factors, such as teacher-student relationship, flourishing, and academic performance (Chamizo-Nieto et al., 2021); academic performance, academic engagement, and psychological capital resources (Martínez et al., 2019); academic buoyancy, adaptability, and engagement (Martin et al., 2017); and achievement goals, achievement emotions, and academic engagement (Putwain et al., 2022). Despite the growing body of literature that indicates a complex interplay between personal and social-contextual factors, however, most of the studies on the influencing factors of academic engagement have been identified and recognized in the outcome variables such as academic achievement, life satisfaction, and mental health. Among them, academic engagement is mainly regarded as an independent variable, a mediating variable, or a moderating variable, and few studies directly consider academic engagement as a dependent variable. Furthermore, the simultaneous effects were found mainly in the field of Primary, Secondary, and high-school contexts, but less attention has been paid to university settings. Thus, there is a need to further investigate whether or not the patterns of relationships found in previous literature are also found at the university level.

In a sample of university students, the present study would take academic engagement as a dependent variable, in line with previous studies from the field of positive psychology, focusing on individual psychological factors that affect academic engagement and its influencing mechanism. Among all these variables, basic psychological needs satisfaction (BPNS) and emotional intelligence (EI) would be examined simultaneously. To the best of our knowledge, the joint interaction of BPNS with EI abilities to explain academic engagement has not been tested in university students. The identification of these associations allows for a more reliable understanding of how the motivational variables and individual psychological resources are related to the academic engagement with university students. This understanding will provide the basis for the implementation of programs that help to improve academic engagement in the university environment.

### BPNS and academic engagement

Academic engagement describes the degree to which students engage themselves in learning-related activities. Traditionally, academic engagement is considered a multidimensional construct, comprising behavioral, cognitive, and emotional dimensions (e.g., Fredricks et al., 2004; Appleton et al., 2008; Pietarinen et al., 2014). More recently, some studies have articulated a fourth dimension, i.e., agentic engagement, which highlights student’s constructive contribution toward the flow of the instruction he receives (Reeve, 2013).

Due to the following two reasons, the present study used Schaufeli et al.’s definition of engagement, in which academic engagement is described as a “positive, fulfilling, work-related state of mind that is characterized by vigor, dedication, and absorption” (Schaufeli et al., 2002, p. 74). The first reason is that the Schaufeli et al.’s model was proposed directly from university students based on their research on employee engagement. The second reason is that, compared with other educational phase, although university students are not formally employed by the university, students’ studies are involved in coercive, structured tasks and activities (e.g., attending class, cooperating and competing with others, and submitting assignments), which makes academic engagement in university more similar with work (Walker et al., 2006). For example, Saleem et al. (2022) recently posited that post-graduate student’ s educational journey is much different than high-school students in terms of length of the degree, course work, examination, assessment, educational outcomes, teaching methodology, and so on.

According to Schaufeli et al.’s model, academic engagement is a highly motivated and satisfying mental state. Given the motivational nature of academic engagement, a lot of scientific literature in the field of education have investigated the motivational predictors of academic engagement, such as self-efficacy, achievement emotions, and achievement goals (Patrick et al., 2007; Diseth et al., 2012; Song et al., 2015; Stahlberg et al., 2019; Zysberg and Schwabsky, 2021). Among the variables, according to SDT, the fulfillment of the basic psychological needs (i.e., autonomy, competence, and relatedness) is critical to students intrinsic motivation, as these three basic needs are the necessary conditions for individual psychological growth, internalization, and mental health (Jang et al., 2012). For example, in a sample of 648 university teachers in China, Jin et al. (2022) found that meeting the three types of BPNS correlated positively with work engagement. Furthermore, a large number of empirical studies have shown that the satisfaction of the three psychological basic needs-either separately or in combination-could have a positive and significant impact on learning engagement and academic performance (Hofer and Busch, 2011; Madjar et al., 2013; Carmona-Halty et al., 2019; Martin and Collie, 2019). For example, in a sample of 366 Korean high-school students using a three-wave longitudinal research, Jang et al. (2016) found that students tend toward a semester-long trajectory of rising engagement when they perceive their teachers to be autonomy supportive and need satisfying. Similarly, Carmona-Halty et al. (2019) also found that students whose basic psychological needs are satisfied at school experience more academic psychological capital, which, in turn, leads to better academic performance.

Furthermore, empirical studies have linked discrete psychological needs to learning and achievement. In a study conducted by Gasiewski et al. (2012) with 2,873 students across 15 Colleges and universities, it was indicated that students who reported feeling comfortable asking questions in class and seeking out tutoring, i.e., autonomy need was respected and satisfied, tended to be more engaged in courses where the instructor persistently displayed an openness to student questions. Regarding to the competency need, which concerns the feeling that individuals experience the control of their environment and ability development. Based on competence motivation theory, Wong et al. (2002) identified self-worth as a significant predictor of motivational orientation and academic outcomes. Related need refers to the feelings of being connected to others. Studies have identified that a supportive teacher-student relationship may provide students with a sense of security that promotes their free and active participation in classroom academic activities (Quin, 2017). More recently, a meta-analysis conducted by Roorda et al. (2017), based on 99 studies with preschool to high-school students, has shown that the total effect size for the associations between both positive teacher-student relationships and engagement was *r* = 0.39 (*p* < 0.01) and negative relationships and engagement was *r* = −0.32 (*p* < 0.01).

Considering past empirical research herein described, this study will propose the following hypotheses:

*Hypothesis 1 (H1)*: BPNS is positively correlated with academic engagement.

### The mediating role of EI

Engagement is not only highly motivated state, but also strongly affected by emotions, which are an inherent part of the human existence in any context (Brackett et al., 2021). Thus, while examining intrinsic factors, researchers also have found emotional intelligence (EI) to be an important predictor of academic engagement (e.g., Sinclair et al., 2003; Mavroveli et al., 2009; Durlak et al., 2011; Huang et al., 2022).

In the scientific literature EI was usually defined in two different ways, i.e., ability model and trait model (Zeidner et al., 2008). Both models have been used in many domains, such as nursing, teaching, physical activity, and teleworking (Law et al., 2004; Smitha et al., 2009; Cebrian et al., 2020). In the current study, EI is conceptualized from the ability model developed by Mayer et al. (2008), which is defined as a mental ability for perceiving, understanding, regulating, and using one’s own and others’ emotions in thinking and action. Research literature suggests that emotionally intelligent people with higher EI also show more positive mood, higher levels of life satisfaction, well-being, flourishing, better psychological adjustment, and lower levels of psychological stress across different samples (e.g., Chu, 2010; Su and Reeve, 2011; Szczygieł and Mikolajczak, 2017; Mérida-López and Extremera, 2020; Karapetyan, 2021).

In the field of education, there is an increasing consensus on the idea that EI is an important skill that teachers and students must develop (Sha et al., 2022). For example, with a total of 702 teachers working at different educational levels in southern Spain, Mérida-López et al. (2020) found that EI and self-efficacy were positively related to teachers’ work engagement and negatively related to withdrawal intentions. As to students, as MacCann et al. (2011) suggest that EI is characterized as the third most important predictor after Intelligence and Conscientiousness in academic achievement. Recently, in a study conducted by Estrada et al. (2021) with 550 students from four higher education institutions and one secondary school, it was observed that EI was shown to be positively related to compassion and higher levels of commitment, which, consequently, led to better academic performance.

Furthermore, some previous research also suggested that EI would positively relate to BPNS (Emery et al., 2016). According to the SDT, psychological needs contain both cognitive and affective elements and BPNS appears as an important motivation factor for the development of EI (Raufelder et al., 2016). For example, in a study conducted with 16 Coaches and 171 youth athletes by Watson and Kleinert (2019), it was observed that coaches’ EI was related to basic need satisfaction in athletes. More recently, in a sample of 1,332 students in Southwest Spain, Rivera-Pérez et al. (2021) suggested that positive and significant associations were found between cooperative academic and EI in all school stages. The results indicated that people with higher BPNS would tend to develop a better EI.

In addition, the mediating role of EI between individual variables and positive psychological outcomes has been proposed in the field of workplace (e.g., Côté, 2014; De Clercq et al., 2014; Castillo-Gualda et al., 2019). For example, in a sample of 201 Italian workers, Di Fabio et al. (2018) indicated that EI mediated the relationship between personality traits and both hope and optimism. Similarly, the role of individual differences on the strength of implicit motives in the relationship between needs for relatedness and well-being has also been observed (Di Fabio and Kenny, 2016). For instance, in a study conducted by Callea et al. (2019) with 216 Italian participants, it was observed that those who showed the higher levels of psychological need for relatedness were more positively associated with both happiness and flourishing and that EI mediated these associations.

Taken together, based upon past empirical studies and the current knowledge on the role of EI herein described, the present study will propose the following two hypotheses:

*Hypothesis 2 (H2)*: BPNS is positively correlated with Emotional intelligence (EI).

*Hypothesis 3 (H3)*: EI plays a mediating role in the relationship between BPNS and academic engagement.

### The present study

Based on previous findings, this research aimed to investigate relationships among BPNS, EI, and academic engagement in a sample of university students; specifically, the mediated role of the different dimensions of EI would be examined. The present study may contribute to the literature in two ways. First, no studies have simultaneously considered the relationships of both BPNS and EI on academic engagement. By identifying the indirect effects of BPNS on increasing academic engagement as mediated through EI, our results can contribute to the positive psychology literature. Once the relationships were identified, effective interventions could be designed to improve academic engagement among university students.

Second, although the mediated role of EI between individual variables and positive psychological outcomes had been found in the field of workplace, as to which dimension of EI having the mediated role was still a controversial issue (Extremera et al., 2020). By assessing the independent roles of different dimensions of EI between BPNS and academic engagement, the present study may shed some light on the importance of considering EI skills as potential mediated factors in the associations between academic engagement and its correlates.

## Materials and methods

### Participants and procedure

The present study conducted a questionnaire survey at a single university in Zhoukou city from middle China. Students were recruited by means of convenience sampling. This research was conducted in accordance with the Declaration of Helsinki, written informed consent was given to all participants and their privacy, feelings, and intentions were fully considered (Goodyear et al., 2007). With necessary guidance and support, participants voluntarily filled in the questionnaire at the classroom. The electronic Questionnaires were distributed on the spot, and questionnaires would not be submitted until all questions had been answered. Thus, there were no uncompleted questionnaires, and a total of 506 questionnaires were collected. The questionnaire took about 15 min, so questionnaires that took too short, i.e., less than 5 min, or significantly inconsistent, were excluded. Finally, we got 466 valid questionnaires for final analysis. While questionnaire survey was collected, as senior students were on an off-campus internship, they were not included in the sample. Among participants, 88 were men (18.9%) and 378 were women (81.1%), with a predominantly female student sample. Overall, there were 332 first-year students (71.2%), 111 s-year students (23.8%), and 23 juniors (4.9%). As to major, there were 308 students (66.1%) in arts and social science, 66 students (14.2%) in science, and 92 students (19.7%) in engineering.

### Measures

#### Academic engagement scale

Academic engagement was assessed by the Work Engagement Student Scale (UWES-SS) created by Schaufeli et al. (2002). The scale consists of 14 items which evaluate three dimensions of academic engagement: (1) vigor, with 5 items (e.g., When studying I feel strong and vigorous); (2) dedication, with 5 items (e.g., My studies inspire me); and (3) absorption, with 4 items (e.g., I can get carried away by my studies). All items were scored on a five-point Likert scale, from 1 (never) to 5 (always). A higher score on this scale indicates a higher level of academic engagement.

#### Basic psychological needs satisfaction scale

Basic Psychological needs satisfaction was assessed with Basic Psychological Needs Satisfaction scales (BPNSs), proposed by Ryan and Deci (2017). The scale consists of 14 items which evaluate three dimensions of BPNS: (1) Autonomy, with 6 items (e.g., I can try to solve tasks my own way); (2) Competence, with 6 items (e.g., I am considered capable of difficult tasks); and (3) Social relatedness, with 4 items (e.g., I feel accepted by my classmates). This scale adopts a 5-point Likert scoring system, from 1 (strongly disagree) to 5(strongly agree). A higher score on this scale in university students indicates a higher level of the satisfactions of their basic psychological needs.

#### Emotional intelligence scale

Emotional intelligence was assessed with the Wong and Law Emotional Intelligence Scale (WLEIS). This scale is a self-report measure, composed of 16 items, with a 5-point Likert-type scale ranging from 1 (strongly disagree) to 5 (completely agree). The Chinese validation (Sha et al., 2022) proposed a factor solution in four dimensions: (1) Self-emotion appraisal (SEA), with 4 items (e.g., I really understand what I feel); (2) Others’ emotion appraisal (OEA), with 4 items (e.g., I am a good observer of others’ emotions); (3) Use of emotion (UOE), with 4 items (e.g., I am a self-motivated person); and (4) Regulation of emotion (ROE), with 4 items (e.g., I have good control of my own emotions). A higher score on this scale indicates a greater degree of emotional intelligence.

#### Demographic variables

Participants’ demographic data, including information about gender, grade level, and major, were collected with our questionnaire. Previous research showed that those demographic variables may have a direct or mediated effect on basic Psychological needs satisfaction and emotional intelligence (Madjar et al., 2013; Martin and Collie, 2019; Cebrian et al., 2020; Karapetyan, 2021), so they were considered as controls in the present study.

### Reliability and validity analysis

To evaluate the measurement model, the following indicators were used in the present study: standardized factor loadings, component reliability (CR), average variance extracted (AVE), Cronbach’s alpha coefficients (*α*), and discriminant validity. The results of reliability and validity analysis are shown in Table 1.

**Table 1:** Results of reliability and validity analysis.

Variables	FL	CR	AVE	Cronbach’ *α*
Basic psychological needs satisfaction				0.860
Autonomy	0.531–0.834	0.793	0.440	
Competence	0.504–0.648	0.677	0.346	
Social relatedness	0.577–0.914	0.844	0.585	
Emotional intelligence				0.830
Self-emotion appraisal	0.508–0.830	0.799	0.507	
Others’ emotion appraisal	0.743–0.915	0.893	0.678	
Use of emotion	0.554–0.836	0.807	0.516	
Regulation of emotion	0.717–0.864	0.868	0.623	
Academic engagement				0.899
Vigor	0.703–0.796	0.838	0.565	
Dedication	0.706 –0.817	0.826	0.544	
Social relatedness	0.681–0.779	0.768	0.526	

FL, factor loadings; CR, component reliability; AVE, average variance extracted.

At the beginning, item analysis was used to eliminate inappropriate questions from the questionnaire. There were totally 46 items in the original questionnaire, the items with factor loadings below 0.5 were deleted. After this process, three items in the Basic Psychological Needs Satisfaction Scale (i.e., “we are taught to work independently” in the Autonomy Subscale, “I have already learned a lot with this teacher” and “I feel challenged in class” in the Competence Subscale), and three in the Academic Engagement Scale (i.e., “When I get up in the morning, I feel like going to class” in the Vigor Subscale, “I find my studies challenging” in the Dedication Subscale and “Time flies when I′ m studying” in the Absorption Subscale), the factor loadings of which were below 0.5, were deleted. Finally, 40 items in total remained for further analysis, including 11 for the Academic Engagement scale (i.e., 4 items in the Vigor Subscale, 4 items in the Dedication Subscale, and 3 items in the Absorption Subscale), 16 for the Emotional intelligence scale (i.e., 4 items in the Self-emotion appraisal Subscale, 4 items in the Others’ emotion appraisal Subscale, 4 items in the Use of emotion Subscale, and 4 items in the Regulation of emotion Subscale), and 13 for the Basic Psychological Needs Satisfaction scale (i.e., 5 items in the Autonomy Subscale, 4 items in the Competence Subscale, and 4 items in the Social relatedness Subscale). As Table 1 illustrates, the factor loadings of all instrument items were all above 0.5.

In addition, results showed that all CR values were greater than 0.6, and all AVE values, except autonomy and competence items, were greater than 0.5. Even though the AVE is less than 0.5, according to Fornell and Larcker (1981), if the CR value exceeds the criteria of 0.6, the scale’s convergent validity is still acceptable. Meanwhile, internal consistency reliability (Cronbach’s alpha) exceeded 0.8 for all items, indicating good reliability of this study’s constructs.

Finally, the square root of AVE was performed to examine the discriminant validity of all the research instruments. If the square root value of AVE was greater than the correlation coefficient in each dimension (Fornell and Larcker, 1981), the discriminant validity of the constructs was suitable. As shown in Table 2, the results met the criteria for assessing discriminant validity.

**Table 2:** Discriminant validity of the research instruments.

Variables	BPNS-A	BPNS-C	BPNS-SR	EI-S0E	EI-OEA	EI-UOE	EI-ROE	AE-V	AE-D	AE-A
BPNS-A	**0.664**									
BPNS-C	0.602***	**0.588**								
BPNS-SR	0.345***	0.499***	**0.765**							
EI-SEA	0.235***	0.220***	0.123**	**0.712**						
EI-0EA	0.119*	0.131**	0.145**	0.323***	**0.823**					
EI-UOE	0.339***	0.471***	0.375***	0.264***	0.133**	**0.718**				
EI-ROE	0.153**	0.158**	0.202***	0.233***	0.152**	0.240***	**0.789**			
AE-V	0.281***	0.354***	0.299***	0.135**	0.083	0.445***	0.175***	**0.752**		
AE-D	0.355***	0.400***	0.269***	0.236***	0.085	0.515***	0.137**	0.687***	**0.738**	
AE-A	0.323***	0.354***	0.254***	0.161***	0.096*	0.438***	0.182***	0.579***	0.586***	**0.725**
M	3.517	3.439	3.583	3.701	3.513	3.491	3.334	3.082	3.307	3.137
SD	0.515	0.494	0.576	0.557	0.617	0.608	0.640	0.606	0.690	0.690

***p < 0.001. M, mean; SD, standard deviation; BPNS-A, autonomy; BPNS-C, competence; BPNS-SR, Social relatedness; EI-SEA, Self-emotion appraisal; EI-OEA, Others’ emotion appraisal; EI-UOE, Use of emotion; EI-ROE, Regulation of emotion; AE-V, vigor; AE-D, dedication; AE-A, absorption. Bolded fonts are AVE square root values.

### Data analysis

The data were analyzed using the SPSS (version 21.0) software. The first analysis consisted of descriptive statistics (including mean and standard deviation) and Pearson’s correlations among BPNS, EI, and academic engagement (Hayes, 2013). Then, the SPSS Amos program (version 21.0) was used to evaluate the mediating effect of EI on BPNS and academic engagement. The present study is based on the structural equation modeling (SEM) technique, which is a nominal research analysis approach (Saleem et al., 2022). For the mediation analyses, a bootstrapping method was further used to obtain bias-corrected 95% confidence intervals (95% CI) with 5,000 re-samples. If the 95% CI did not contain zero, an effect was considered significant.

## Results

### Common method bias

As all variables used in the present study were measured by a self-report questionnaire, there may have a common method deviation. Before analysis, Harman’s single-factor test was used (Podsakoff et al., 2003). In the present study, there were 10 factors with feature values greater than 1 extracted. The explanatory variance of the first factor was 23.738%, less than the 50% threshold. Thus, the common method deviation of data in the present study was not serious.

### Descriptive statistics and correlation analysis of main variables

Prior to the assessment of the hypotheses, descriptive statistics and Pearson’s correlations among variables were conducted. As shown in Table 3, BPNS was positively related with academic engagement (*r* = 0.458). As expected, BPNS correlated significantly with the four dimensions of EI (SEA: *r* = 0.240; OEA: *r* = 0.163; UOE: *r* = 0.482; ROE: *r* = 0.212). In the same way, the four subscales of EI were also positively correlated with academic engagement (SEA: *r* = 0.209; OEA: *r* = 0.101; UOE: *r* = 0.542; ROE: *r* = 0.187). In addition, the correlation coefficients between the variables range from 0.101 to 0.542, less than 0.700, indicating that there is no serious collinearity between the three variables.

**Table 3:** Descriptive statistics and correlations among the study variables.

Variables	M	SD	BPNS	SEA	OEA	UOE	ROE	AE
BPNS	3.513	0.425	1					
SEA	3.701	0.557	0.240***	1				
OEA	3.513	0.617	0.163***	0.323***	1			
UOE	3.491	0.608	0.482***	0.264***	0.133**	1		
ROE	3.334	0.640	0.212***	0.233***	0.152**	0.240***	1	
AE	3.179	0.571	0.458***	0.209***	0.101*	0.542***	0.187***	1

***p < 0.001. M, mean; SD, standard deviation; BPNS, basic psychological needs satisfaction; SEA, Self-emotion appraisal; OEA, Others’ emotion appraisal; UOE, Use of emotion; ROE, Regulation of emotion; AE, academic engagement.

### Measurement and structural model

We assessed both measurement and structural models for all of the study variables, i.e., BPNS, EI, and academic engagement through different fitness indexes such as absolute, incremental, and parsimonious fit indices (Hu and Bentler, 1999). Per the guidelines, measures like normed chi-square (*X*^2^/df); RMR (root mean residual); RMSEA (root mean square error of approximation); CFI (comparative fit index); GFI (goodness of fit index); TLI (Tucker-Lewis index); IFI (incremental fit index); and SRMR (standardized root mean residual) were utilized (Schweizer, 2010). The measurement model and structural model outcomes are shown in Table 4. While performing a confirmatory factor analysis of the scale, an excessive sample size may cause the increased Chi-square values; therefore, other adaptation indicators were considered the model fit (Hu and Be Ntler, 1998; Hsiao et al., 2015). Overall, the results revealed a good fit of the measurement model for each scale in the present study.

**Table 4:** Measurement model and structural model validity.

	*X*^2^/df	RMR	RMSEA	CFI	GFI	TLI	IFI	SRMR
BPNS	4.587	0.032	0.088	0.904	0.912	0.879	0.904	0.064
EI	2.365	0.021	0.054	0.961	0.939	0.952	0.961	0.039
SEA	6.650	0.015	0.110	0.981	0.986	0.943	0.981	0.031
OEA	4.020	0.007	0.081	0.995	0.991	0.984	0.995	0.014
UOE	6.909	0.016	0.113	0.980	0.985	0.941	0.981	0.030
ROE	28.531	0.025	0.243	0.942	0.938	0.825	0.942	0.044
AE	2.859	0.025	0.063	0.967	0.953	0.956	0.967	0.037
Structural model results	2.511	0.039	0.057	0.936	0.907	0.926	0.936	0.075

*X^2^*/df, normed Chi-square; RMR, root mean residual; RMSEA, root mean square error of approximation; CFI, comparative fit index; GFI, goodness of fit index; TLI, Tucker-Lewis index; IFI, incremental fit index; SRMR, standardized root mean residual; BPNS, basic psychological needs satisfaction; EI, emotional intelligence; SEA, Self-emotion appraisal; OEA, Others’ emotion appraisal; UOE, Use of emotion; ROE, Regulation of emotion; AE, academic engagement.

### Hypothesis testing

**Figure 1:**
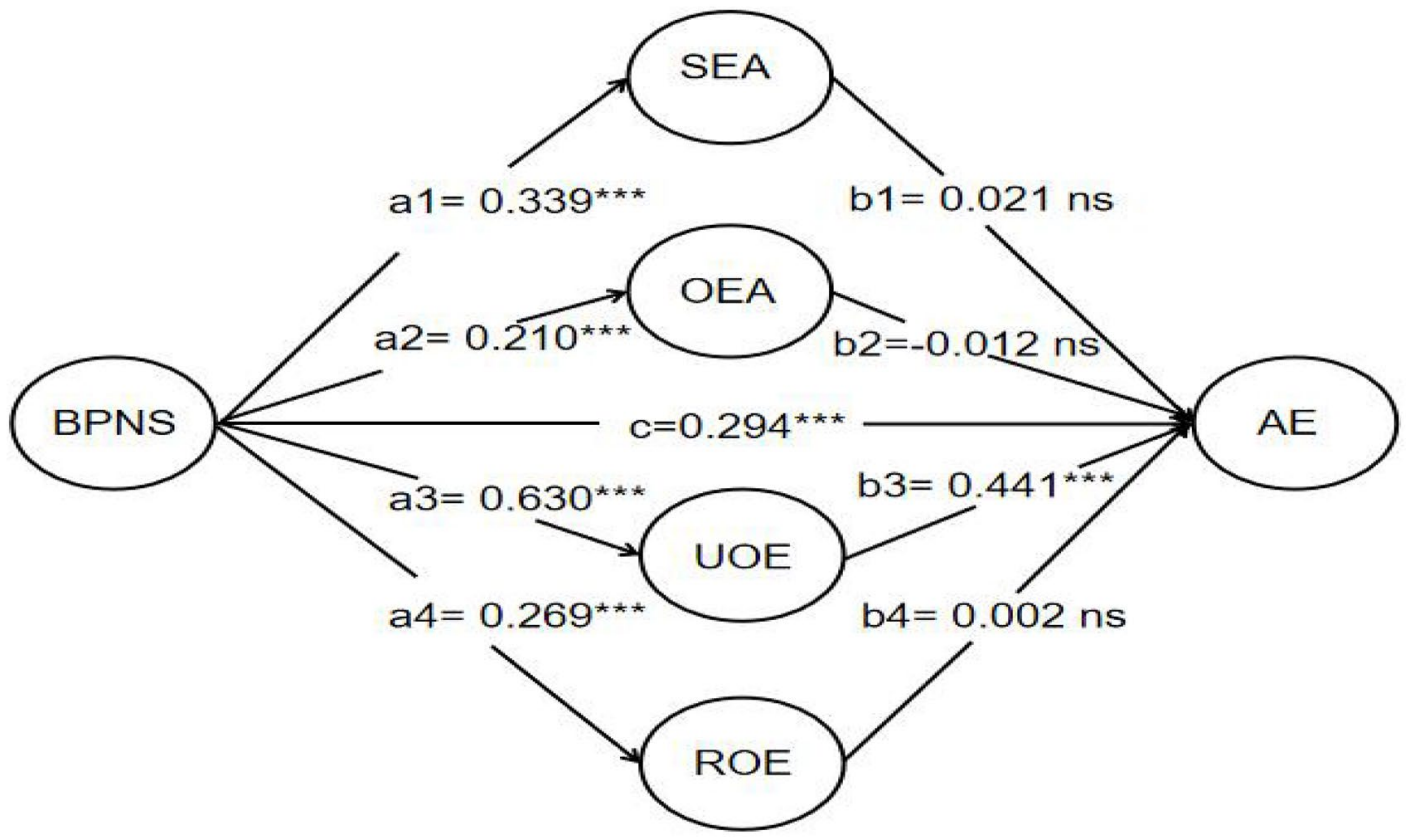
Single mediation model shows the effect of BPNS on AE through the dimensions of EI. Standardized coefficients are presented. BPNS, basic psychological needs satisfaction; AE, academic engagement; SEA, Self-emotion appraisal; OEA, Others’ emotion appraisal; UOE, Use of emotion; ROE, Regulation of emotion.***p<0.001; ns, non-significant effect.

To analyze the influence of BPNS on academic engagement and the role of EI, SEM approach was used, the results are listed in Figure 1. Specifically, these results showed that university students’ BPNS significantly predicted academic engagement (*β* = 0.294, *p* < 0.001). Therefore, the research hypothesis H1 was supported. The second hypothesis H2 (BPNS is positively correlated with Emotional intelligence on EI) also was observed. The standardized coefficient of BPNS on SEA was *β* = 0.339 (*p* < 0.001), on OEA was *β* = 0.210 (*p* < 0.001), on UOE was *β* = 0.630 (*p* < 0.001), and on ROE was *β* = 0.269 (*p* < 0.001). That is, the research hypothesis H2 was supported. We also tested the mediated effect of four dimensions of EI between BPNS and academic engagement, the results showed BPNS still has a significant positive effect on academic engagement. However, three of the mediators, i.e., SEA, OEA, and ROE, had non-significant effects on academic engagement, only one dimension of EI, that is UOE, had a significant positive effect on academic engagement (*β* = 0.441, *p* < 0.001). Therefore, the research hypothesis H3 was supported, i.e., it was the UOE dimension of EI that played a partially mediating role in the relationship between BPNS and academic engagement.

### Test of mediation

In order to further test the mediating effect, the bootstrap method was used in a procedure of 5,000 re-samples to analyze the mediating effects *via* the SPSS Amos program. In this process, a 95% confidence interval (95% CI) would be generated to test the significance of the indirect effect between BPNS and academic engagement through mediating roles of the four dimensions of EI. If zero does not exist between the lower and upper confidence intervals, the direct effects would be identified as significant. Table 5 shows the Bootstrapping analysis results.

**Table 5:** Test of multiply mediation effects.

The path	Effect of value	Bootstrap 95% CI	
		Lower limit	Upper limit
Standardized direct effect			
BPNS→SEA	0.339*	0.216	0.459
BPNS→OEA	0.210*	0.088	0.326
BPNS→UOE	0.630*	0.521	0.724
BPNS→ROE	0.269*	0.143	0.388
BPNS→AE	0.294*	0.128	0.455
SEA→AE	0.021	−0.084	0.130
OEA → AE	−0.012	−0.122	0.099
UOE → AE	0.441*	0.278	0.602
ROE→AE	0.002	−0.097	0.106
Standardized Indirect effect			
BPNS→UOE → AE	0.283*	0.183	0.411
Standardized total effect			
BPNS→AE	0.577*	0.479	0.674

Bootstrapping random sampling 5,000 times. CI; confidence interval; BPNS, basic psychological needs satisfaction; EI, emotional intelligence; AE, academic engagement; SEA, Self-emotion appraisal; OEA, Others’ emotion appraisal; UOE, Use of emotion; ROE, Regulation of emotion.

As shown in Table 5, the direct effect value of BPNS on academic engagement was 0.294, accounting for 50.95% of the total effect, and the 95% CI was [0.128, 0.455], indicating that the direct effect was significant. After including the four dimensions of EI as mediators in the process, the total indirect effect was still significant, but only the dimension of UOE served as a significant mediating variable. The indirect effect value of UOE on BPNS and academic engagement was 0.283, accounting for 49.04% of the total effect, with a 95% CI [0.183, 0.411]. Therefore, these results indicated that not all dimensions of EI, but only UOE dimension, played a partially mediating role in the effect of BPNS on academic engagement.

## Discussion

### Main findings

This research aimed to determine the BPNS-academic engagement association in university students and the mediating effects of EI abilities. The results of the present study showed that BPNS could positively affect academic engagement through EI abilities.

Firstly, consistent with findings in previous psychology studies, the correlation suggested that both basic psychological needs and emotional intelligence positively related to university students’ academic engagement. On the one hand, this finding is consistent with self-determination theory, i.e., satisfying all three needs is critical for psychological well-being, in turn, promoting mental health and significantly impacting engagement. As previous studies suggest, when students report a high perception of autonomy support from teachers, they view their class activities as volitional and self-determined and engage in class willing and eagerly (e.g., Madjar et al., 2013; Martin and Collie, 2019). The positive effects associated with BPNS allow university students to be more energized, proactive, and motivated, and these positive mental states as resources could enhance the higher levels of engagement. On the other hand, the correlation results are also in line with the JD-R theory, previous research mainly focused on the relationship between EI abilities and individual and work-related well-being domains, such as job and life satisfaction in teachers, in nurses, and in healthcare (e.g., Law et al., 2004; Smitha et al., 2009; Cebrian et al., 2020). In line with this, this study further extended the model to university students.

In this sense, our study is coherent with the need to return to a more humanistic education that incorporates a new language and new content. Added to this perspective is the need to incorporate emotions into the teaching-academic process and give them a greater role by establishing the relationship between emotional factors and motivational reasons as an additional way of strengthening and developing the individual academic variables. When the needs of students are satisfied or fulfilled with contextual support, students also show higher levels of EI, and these interpersonal and psychological reciprocal effects could positively predict the engagement.

Secondly, the results showed that Hypothesis 2 was supported, stating that BPNS would positively relate to EI, which would add new light on the relationship between motivational systems and EI (Bechter et al., 2021). Despite the mediated role of motivational variables and individual psychological resources had been examined, respectively, in current scientific literature, the explicit role of emotions in the motivation-generative mechanism was still poorly investigated. Recently, some research had explored the issue among different populations. For example, Watson and Kleinert (2019) showed that coaches’ EI was related to basic need satisfaction in athletes. More recently, in a study conducted by Callea et al. (2019) with 216 Italian participants, suggested that the need for relatedness would positively relate to EI.

In line with those studies, the results obtained from this study could support a new evidence in the relationship between BPNS and EI, indicating that people with higher levels of BPNS will tend to develop a better EI. Thus, it is possible to envisage that the most emotionally intelligent people who are satisfied in basic psychological needs display higher levels of individual well-being. That is, university students who show higher levels of BPNS are not only more motivated and full of energy, but also more emotionally intelligent. In turn, being more emotionally intelligent did increase the levels of academic engagement.

Finally, the present study also seemed to support Hypothesis 3, which stated that the contribution of BPNS to academic engagement would be mediated by EI abilities, but only through the UOE dimension of EI abilities among Chinese university students. These findings are consistent with previous work that show the mediating effect of EI between personal resources and work engagement/outcomes. However, as to which dimension of EI abilities may work in enhancing positive mental state within the JD-R theory still remains unclear. Among these, the often mentioned dimension of EI was Emotion regulation ability (ERA) and/or UOE. For example, a study conducted by Mérida-López and Extremera (2020), with 190 teachers in Spain, found that only ERA was significantly associated with work engagement, job satisfaction, and life satisfaction. Other studies found both ERA and UOE of EI could play the mediated role. In a study with 380 Chinese adults, Bao et al. (2015) showed that mindfulness was positively associated with four components of EI abilities, and negatively associated with perceived stress. Additionally, the regulation and use of emotion components of EI could act as mediators of the association between mindfulness and perceived stress.

Notwithstanding this, the present study did not provide evidence to consider the dimension of ROE as a mediator in the link to EI performance. Compared with other dimensions, the results suggested that only the dimension of UOE could play a significant mediating role between BPNS and academic engagement. The finding was in line with a study more recently carried out by Parent-Lamarche (2022). Based on a sample of 254 Canadian employees from 18 small and medium organizations during the COVID-19 pandemic, Parent-Lamarche (2022) found that except for skill utilization and recognition, use of emotion could appear to be key considerations for organizations that wish to increase work engagement and decrease intention to quit.

The reason why it was UOE not ROE that played the mediating role in relationships between BPNS and academic engagement may be inferred from the essential characteristics of both. Judging from the essential characteristics of ROE, it emphasizes the capability to control and regulate emotions, which is conductive to relieving and mitigating one’s psychological distress, recovering from negative situations, and adjusting their emotions flexibly (Cheung and Ng, 2019). However, judging from the essential characteristics of UOE, it mainly focuses on using and maximizing existing individual psychological resources, and it is of importance to enhancing one’s psychological functionings and well-being, which is crucial for improving positive state and outcomes (Chen et al., 2020). From the perspective of positive psychology, once the basic psychological needs were satisfied, several beneficial academic outcomes, such as the achievement of positive emotions at school (e.g., joy, interest, contentment, and school satisfaction), and more effort would be activated. These emotions, based on the broaden-and-build theory (Fredrickson, 2001), may further broaden students’ momentary thought-action repertoires and build their enduring personal resources. In this respect, using the emotions produced from BPNS could help students to open their minds to different thoughts and problem-solving approaches, and, in turn, enhance academic engagement.

Like other research, the results from here are not conclusive; hence, the need for more research should be added up so as to understand this issue.

### Limitations and future research

Some potential limitations should be mentioned in this study. First, the present research used a cross-sectional questionnaire, so the findings may have been affected by unpredictable social interactions, which prevents us from drawing causal conclusions. In order to fully disentangle reciprocal causal relations, an additional, longitudinal design study is required in the future. Second, there is a limitation directly connected to how EI is measured. In this study, the WLEIS is a self-report instrument. Although the scale has been shown to be both reliable and valid, it is recommended to use both self-reports and performance tests to measure EI (Brackett et al., 2006). Third, the sample included only university students in China, so the results cannot be generalized. To further test the findings, future studies should incorporate participants from other cultures and other countries to form a bigger and more representative sample.

### Practical implications

Despite these limitations, the present results have crucial implications for teachers and university administrators. First, considering the direct effect of BPNS on academic engagement, autonomy-supportive learning environments are of great significance of teachers to foster students’ positive mental state. For instance, teachers should carry out certain instructional behavior, such as attending to the students’ perspective, vitalizing inner motivational resources, appreciating and accepting negative affect, and displaying patience (Diseth et al., 2018). More recently, one intervention named Peer Assisted Study Sessions (PASS), a structured peer-led study group where students collectively share knowledge and solve course-related tasks, both cross-sectional and longitudinal analyses showed its positive outcomings to academic engagement and performance (Rivera-Pérez et al., 2021).

Second, although scholars and administrators acknowledge the importance of EI to academic engagement and achievement, a lot of intervention techniques aimed at improving the students’ EI have been designed (Vesely et al., 2013; Mérida-López et al., 2021), most training programs were mainly focused on developing the emotional skills of students, especially the abilities of emotional regulation and management, so as to cope with the problems of academic burnout, pressure and test anxiety. All these measures are necessary to improve students’ academic engagement, but not sufficient, at least when positive psychological resources already exist. The results from this study suggested that when students basic psychological needs were satisfied, it was the UOE dimension not ROE dimension of EI that enhanced higher levels of academic engagement. Therefore, EI training programs should aim not only to develop the abilities of emotional regulation and management, but also to improve the awareness of one’s emotion, to be an active interest in using and exploring of one’s positive emotions.

## Conclusion

To the best of our knowledge, our study is the first to explore the means by which EI potentiates the power of basic psychological needs satisfaction to enhance academic engagement among university students. That is, university students who showed higher levels of BPNS were more emotionally intelligent. In turn, being more emotionally intelligent would enhance the levels of academic engagement. In sum, the present study paves the way for future research on the importance of EI as a mediator in the relationship between BPNS and academic engagement in the perspective of positive psychology of sustainable development.

## Data availability statement

The original contributions presented in the study are included in the article/supplementary material, further inquiries can be directed to the corresponding author.

## Ethics statement

The studies involving human participants were reviewed and approved by The Ethics Review Committee of Zhoukou Normal University. Written informed consent to participate in this study was provided by the participants’ legal guardian/next of kin.

## Author contributions

MZ and HC: conceptualization, methodology, software, investigation, data curation, writing-original draft preparation, writing—review and editing, and informed consent statement. All authors contributed to the article and approved the submitted version.

## Conflict of interest

The authors declare that the research was conducted in the absence of any commercial or financial relationships that could be construed as a potential conflict of interest.

## Publisher’s note

All claims expressed in this article are solely those of the authors and do not necessarily represent those of their affiliated organizations, or those of the publisher, the editors and the reviewers. Any product that may be evaluated in this article, or claim that may be made by its manufacturer, is not guaranteed or endorsed by the publisher.
